# REPLY FROM AUTHORS: Septal myectomy performed along the “septal band”

**DOI:** 10.1016/j.xjtc.2022.08.013

**Published:** 2022-08-25

**Authors:** Tsuyoshi Yamabe, Jonathan Ginns, Vijay Vedula, Jay S. Leb, Yuichi J. Shimada, Shepard D. Weiner, Hiroo Takayama

**Affiliations:** a Division of Cardiothoracic and Vascular Surgery, New York Presbyterian Hospital, Columbia University Medical Center, New York, NY; b Department of Cardiovascular Surgery, Shonan-Kamakura General Hospital, Kamakura, Kanagawa, Japan; c Department of Cardiology, Heart Hospital of Austin, Austin, Tex; d Department of Mechanical Engineering, Columbia University, New York, NY; e Department of Radiology, New York Presbyterian Hospital, Columbia University Medical Center, New York, NY; f Department of Medicine Cardiology, New York Presbyterian Hospital, Columbia University Medical Center, New York, NY

Reply to the Editor:

**Figure:**
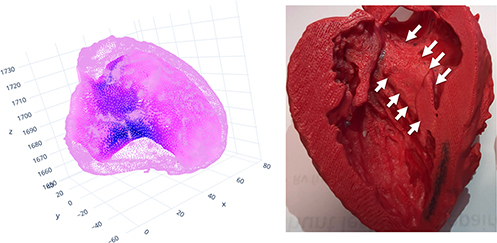

The authors reported no conflicts of interest.

The *Journal* policy requires editors and reviewers to disclose conflicts of interest and to decline handling or reviewing manuscripts for which they may have a conflict of interest. The editors and reviewers of this article have no conflicts of interest.

We appreciate the insightful comments on our paper from Dr Andrushchuk and his colleagues.^1^ First and foremost, we admire their pioneering work on the introduction of 3-dimensional printing technology in septal myectomy.

We agree that left ventricle (LV) remodeling is a concept beyond change in LV wall thickness. Our study showed that the overall LV myocardial volume decreased beyond the extent of resection after septal resection during the relatively short median observation period of 2.5 months. Simultaneously, both LV end-diastolic diameter and end-systolic diameter increased (not statistically significant). With more increase in end-systolic volume relatively to end-diastolic volume, postoperative LV ejection fraction became lower than preoperatively, albeit it remained within the normal range. Previously, such change in ejection fraction was believed to be related to improvement in LV hypercontractile state and diastolic function.^2^ Nonetheless, we need to learn much more from morphology, and we believe surgeons are uniquely equipped to explore this opportunity with the access to advanced imaging and intraoperative assessment.

We also admire the remarkable clinical outcomes of their surgical myectomy. While we believe our outcomes are in line with the literature^3^ and thus satisfactory, we have more to learn. Quality assurance of surgical hypertrophic cardiomyopathy (HCM) repair is not well defined. Given the primary mission of this operation, which is to improve quality of life, ordinal in-hospital outcomes, such as mortality, stroke, and respiratory failure, should not be the main concern. A recent Society of Thoracic Surgeons database study suggests that we need to monitor and improve on other metrics unique to this operation, such as ventricular septal defect, complete heart block, and mitral valve replacement.^4^ We respectfully disagree with negative connotation on mitral valve plasty or repair and instead believe that it is a part of the operation. Addressing abnormal subvalvular tissue and leaflets might be appropriately considered “mitral repair.”^5^ To this point, “surgical HCM repair” instead of “septal myectomy” may better represent what we do. Finally, assessment of the changes in LV outflow tract gradient, systolic anterior motion, and mitral regurgitation is crucial. Here, there are limited data on how much “residual” is clinically acceptable, and this point has a relevance when this operation is compared with other septal-reduction therapies.

**Figure 1:**
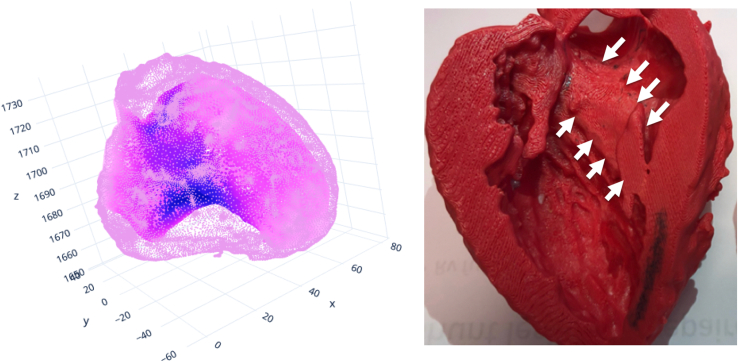
Heat map (*left*) and 3-dimensional model (*right*) of the *left* ventricle showing the septal band (*whitearrows*) in a patient with hypertrophic cardiomyopathy.

The concept of the septal band is fascinating to us. Technically speaking, it may be best visualized through a “heat map,” in which point cloud of the endocardium (average 30,000 points) and the epicardium (average 50,000 points) are depicted using the LV end-diastolic segmentation as well as the wall thickness at each point is color encoded on each point (Figure 1). Based on the observation of our cases with combined basal and midventricular obstruction, we speculate that the mid-ventricular obstruction occurs when the septal band is prominent more toward the apex. In contrast, apical HCM may have a different morphology (although we have observed the band continues to the apex, where it fuses with circumferential apical hypertrophy). Nonetheless, we are eager to continue to learn more from our international colleagues.
